# Prognostic contribution of mammographic breast density and HER2 overexpression to the Nottingham Prognostic Index in patients with invasive breast cancer

**DOI:** 10.1186/s12885-016-2892-y

**Published:** 2016-11-02

**Authors:** Amro Masarwah, Päivi Auvinen, Mazen Sudah, Vaiva Dabravolskaite, Otso Arponen, Anna Sutela, Sanna Oikari, Veli-Matti Kosma, Ritva Vanninen

**Affiliations:** 1 Department of Clinical Radiology, Kuopio University Hospital, Puijonlaaksontie 2, 70210, Kuopio, PO Box PL 100, 70029 KYS Finland; 2 Department of Oncology, Kuopio University Hospital, Puijonlaaksontie 2, 70210, Kuopio, PO Box PL 100, 70029 KYS Finland; 3 Institute of Clinical Medicine, Internal Medicine, University of Eastern Finland, Yliopistonranta 1E, P.O.Box 1627, 70211 Kuopio, Finland; 4 Institute of Biomedicine, Department of Medicine, University of Eastern Finland, Yliopistonranta 1E, P.O.Box 1627, 70211 Kuopio, Finland; 5 Department of Pathology, Kuopio University Hospital, Puijonlaaksontie 2, 70210, Kuopio, PO Box PL 100, 70029 KYS Finland; 6 Biocenter Kuopio and Cancer Center of Eastern Finland, University of Eastern Finland, Yliopistonranta 1E, P.O.Box 1627, 70211 Kuopio, Finland; 7 Institute of Clinical Medicine, University of Eastern Finland, Yliopistonranta 1E, P.O.Box 1627, 70211 Kuopio, Finland

**Keywords:** NPI, Breast density, Prognosis, Prediction, Nottingham prognostic index, HER2

## Abstract

**Background:**

To investigate whether very low mammographic breast density (VLD), HER2, and hormone receptor status holds any prognostic significance within the different prognostic categories of the widely used Nottingham Prognostic Index (NPI). We also aimed to see whether these factors could be incorporated into the NPI in an effort to enhance its performance.

**Methods:**

This study included 270 patients with newly diagnosed invasive breast cancer. Patients with mammographic breast density of <10 % were considered as VLD. In this study, we compared the performance of NPI with and without VLD, HER2, ER and PR. Cox multivariate analysis, time-dependent receiver operating characteristic curve (tdROC), concordance index (c-index) and prediction error (0.632+ bootstrap estimator) were used to derive an updated version of NPI.

**Results:**

Both mammographic breast density (VLD) (*p* < 0.001) and HER2 status (*p* = 0.049) had a clinically significant effect on the disease free survival of patients in the intermediate and high risk groups of the original NPI classification. The incorporation of both factors (VLD and HER2 status) into the NPI provided improved patient outcome stratification by decreasing the percentage of patients in the intermediate prognostic groups, moving a substantial percentage towards the low and high risk prognostic groups.

**Conclusions:**

Very low density (VLD) and HER2 positivity were prognostically significant factors independent of the NPI. Furthermore, the incorporation of VLD and HER2 to the NPI served to enhance its accuracy, thus offering a readily available and more accurate method for the evaluation of patient prognosis.

## Background

Breast cancer is a heterogeneous disease with differing behaviors and responses to therapy [1, 2]. Therefore, many prognostic models have been proposed for investigating patient outcome in relation to multiple patient and disease characteristics and to support clinical decision making. The Nottingham Prognostic Index (NPI) was first introduced in 1982 and has since been validated in independent large multicenter studies with long term follow up [3–6]. It is based on traditional prognostic factors such as tumor size, lymph node status and histological grade. It gives clinicians the ability to predict both the clinical outcome of tumors and the need for systemic therapies.

Mammographic breast density (MBD) refers to the relative abundance of fibrous and glandular tissues compared to the fat content of the breast as they appear on a normal X-ray mammogram. Increased MBD is considered as an established risk factor for breast cancer development [7], while previous studies reported that in patients with already diagnosed breast cancer tumors originating in breasts with very low density (VLD) were shown to be associated with a poorer prognosis even after correcting for possible confounders [8, 9].

Human Epidermal Growth Factor Receptor 2 (HER2) receptor is a membrane tyrosine kinase and is considered as a major driver of tumor development and progression [10]. Patients overexpressing HER2 historically showed a higher recurrence rates and a generally poorer outcome [11], but since the introduction of HER2-directed therapies significant improvements in patients’ outcomes have occurred. Nowadays, several guideline bodies recommend routine testing of HER2 and also adjuvant treatment with trastutsumab in HER2-positive cases [12, 13]. Estrogen receptor (ER) and progesterone receptor (PR) statuses are also well known prognostic and predictive factors and play a key role in breast cancer outcome and treatment [14]. This indicates that the aforementioned factors that are routinely available may also have a role in prediction accuracy enhancement if successfully incorporated into scoring systems such as the NPI.

In this study we set to examine the associations between very low mammographic breast density (VLD), HER2 status, ER and PR status in a homogenized patient group with matched NPI categories. Our main purpose was to assess whether those variables could be added to the NPI to form a new more accurate scoring system with enhanced prognostic and predictive values in order to better detect patients who are at high risk.

## Methods

This study was based on a database of 278 breast carcinoma cases which was prospectively gathered to study the relationship of HER2 status and biological markers. The criteria for patient selection have been described elsewhere [15]. Shortly, 139 consecutive HER2 positive patients who were operated on in our university hospital were collected during the years 2002 – 2008 and matched with an equal amount of HER2 negative breast cancer cases with matching age and time of operation. All pathological, clinical and radiological data were blinded at the time of patient selection with the exception of HER2 status. The permission for this study was provided by the ethics committee of University of Eastern Finland, informed consent for this study was waived by the Finnish National Supervisor Authority for Welfare and Health (VALVIRA).

All available digital mammograms of the patients were then retrospectively collected and the analogue mammograms were digitized and collected into a database. Many of the patients in the study population have been diagnosed and referred from other hospitals and centers from our university hospital’s catchment area which means that multiple mammographic imaging systems have been used to obtain the diagnostic images used in the analyses. The diagnostic mammograms that first revealed the tumors were chosen for the evaluation as described previously [8]. The percentage of the area of the mammogram occupied by radiologically dense breast tissue were assessed using the craniocaudal projections and were determined visually. Eight patients had to be excluded after the initial collection because of unsatisfactory mammograms or missing projections bringing the final number of patients included in the analysis to 270.

All mammograms were first analyzed independently and then in consensus by five trained radiologists (three breast radiology specialists and two residents). The percentage of the area of the mammogram occupied by radiologically dense breast tissue was assessed visually from the craniocaudal projections and then distributed into six different percentile categories (<5, 5–10, 10–25, 25–50, 50–75 or >75 %). For the purpose of this study, density was dichotomized into Very Low Density (VLD; ≤10 %) and Mixed Density (MID; >10 %) to allow the variables to be treated as binary throughout the analysis. The expression of HER2 gene amplification was determined by the chromogenic in situ hybridization test (CISH test) by Zymed SPo-LightTM CISHTM Kit (Zymed 84-0146, San Francisco, CA). Cancers with six or more gene copies were considered as HER2 positive [16].

The NPI was calculated from the available data using the formula: NPI = tumor size (in cm) x 0.2 + histological grade (1–3) + lymph node points (negative node = 1; 1–3 positive node = 2; 4 or more positive node = 3) [17]. NPI was further subdivided into three prognostic categories: 1) -low risk, with NPI equal to or less than 3.4; 2) -medium risk, with NPI between 3.4 and 5.4; 3) -high risk, with NPI over 5.4.

The baseline characteristics of the patients have been presented previously [15] and are presented in (Table 1). The adjuvant treatments were given according to national guidelines which are in accordance with the international guidelines [18–20]. Chemotherapy was provided to 198 patients (73.3 %), hormonal treatment to 172 (63.7 %), while postoperative radiotherapy was given to 240 (88.9 %) patients. Adjuvant trastuzumab was routinely given to all HER2-positive patients from the year 2005 onwards, while before that it was given to select patients participating in a trial [21]. HER2-positive patients received adjuvant trastuzumab in 60 (45.1 %) of the 133 cases. For all events that occurred to patients in our study population, there was no difference in treatment plans between patients according to their dichotomized density profiles (Table 2). Follow up was collected from medical records and is up to date as of October 2014.

**Table 1:** Clinicopathological characteristics of the patients

Characteristic	Number of cases (%)
Patient number	270
Age (Years)	
Mean	58.8
Range	32–86
Postmenopausal (%)	66.3 %
Mean tumor size (mm)	22.73
VLD patients	21.47 (6–60)
MID patients	23.46 (3–90)
Mean BMI	26.70
VLD patients	25.46 (20.24–46.87)
MID patients	28.84 (17.96–41.53)
HER2 positive	133 (49.3 %)
Triple Negative	17
Tumor Pathological T classification	
T1	152 (56.3 %)
T2	95 (35.2 %)
T3	10 (3.7 %)
T4	13 (4.8 %)
Tumor N classification	
N0	100 (37.0 %)
N1	117 (43.3 %)
N2	34 (12.6 %)
N3	19 (7.0 %)
Definitive histology	
Ductal	223 (82.6)
Lobular	26 (9.6 %)
Mucinous	4 (1.5 %)
Other	17 (6.3 %)
Histological grade	
1	22 (8.1 %)
2	120 (44.4 %)
3	128 (47.4 %)
Follow up time / years	
Mean	8.03
Range	0.39–13.22

**Table 2:** The *p* values for the differences in treatment options for patients who died or had a relapse (*n* = 57) according to their dichotomized density profiles

	VLD vs MID*
Adjuvant chemotherapy	0.398
Herceptin	0.229
Hormonal Therapy	0.419
Radiotherapy	0.762

*VLD very low densiy, *MID* Mixed density

### Statistical analysis

Statistical analysis was performed with software (SPSS, version 19; SPSS, Chicago, Ill) and R (version 3.2.0) for Windows. Patients with bilateral disease (*n* = 8) had both breasts analyzed separately, one patient with bilateral disease and conflicting density readings between the breasts was integrated in the analysis by choosing the side with the worse stage and grade. The relationships between MBD, HER2 and NPI were evaluated using cross tabulation and McNemar’s non-parametric paired proportions test. Survival amongst the different patient groups was compared by the Kaplan-Meier method using log rank (Mantel-Cox) test. Univariate analysis was used on different categorical prognostic factors individually and Hazard Ratios (HR) with 95 % confidence intervals were estimated. Cox Multivariate analysis was then used in a backward stepwise manner to assess the factors combined until the best fit was obtained and HR and 95 % CI were recorded. Survival prediction model for breast cancer patients starting with NPI was followed by adding more variables to it to improve it and analyzed by using Cox multivariate analysis, time-dependent receiver operating characteristic curve (tdROC), concordance index (c-index) and prediction error (i.e. 0.632+ bootstrap estimator).

## Results

The average NPI for our patient population was 4.66 (range 2.12–7.40), where 21.5 % (58/270) of patients belonged to the low risk prognostic group, 47.0 % (127/270) belonged to the intermediate risk group and 31.5 % (85/270) to the high risk group. As expected, patients’ disease free survival (DFS) declined with increasing values of NPI ranging from 91.4 % (53/58), 87.4 % (111/127), to 42.4 % (36/85) for patients in the low, intermediate and high risk groups of NPI respectively (*p* < 0.001).

Mammographic breast density, ER and PR statuses were normally distributed between the different NPI groups (*p* = 0.211, *p* = 0.528, *p* = 0.472, respectively). The percentage of HER2 positive patients progressively increased from the low (29.3 %, 17/58), intermediate (47.2 %, 60/127) and to the high risk (65.9 %, 56/85) prognostic groups of NPI (*p* < 0.001).

As mentioned earlier, patients in the intermediate risk group of NPI had a DFS of 87.4 % (111/127). The addition of VLD factor alone (HER2 negative patients) reduced survival to 82.6 % (19/23). The addition of both VLD and HER2 positivity at the same time reduced survival in this intermediate risk category to 70.0 % (14/20). The patients in this category who were both negative for HER2 and had MID breasts had a survival of 93.2 % (41/44), (*p* = 0.02).

In the high risk group of NPI, the DFS was 42.4 % (36/85) as mentioned earlier. The addition of VLD factor alone (HER2 negative patients) reduced survival to 30.0 % (3/10). The addition of both HER2 positivity and VLD simultaneously dropped survival to 10.5 % (2/19). Patients in this high risk category who were both HER2 negative and MID had a relatively better prognosis with a DFS of 63.2 % (12/19), (*p* = 0.001).

In our database, ER and PR statuses had no significant impact on survival in any of the groups of NPI. Unfortunately, the previously described analyses could not be performed in the low risk group due to the low number of patients in this group and the low number of events that have occurred there.

**Fig. 1:**
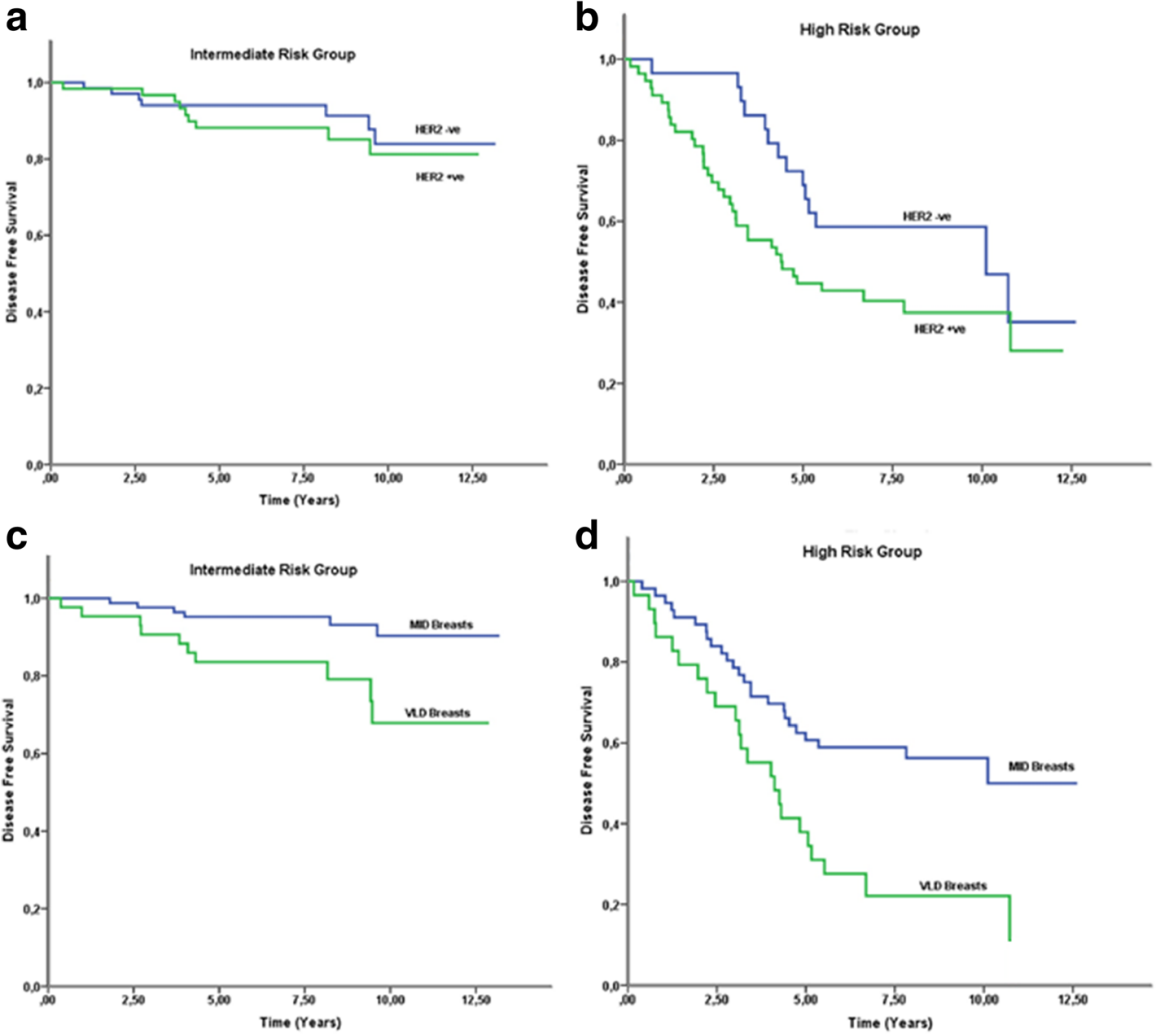
Patients’ Disease free survival graphs according to HER2 status and their MBDs. Graphis depiciting DFS according to patients’ HER2 receptor status (*p* = 0.049) separately for patients in the (**a**) intermediate and (**b**) high risk NPI groups. Disease free survival graphs according to patients’ dichotomized mammographic density values. (*p* < 0.001) separately for patients in the (**c**) intermediate and (**d**) high risk NPI groups

To assess the prognostic powers of those factors in more detail, we evaluated the survival percentages according to the different prognostic groups of NPI. First, as shown in Fig. 1a and b, the DFS for HER2 negative patients was significantly better than for HER2 positive patients in both the intermediate and the high risk groups respectively (89.6 vs 85.0 % and 51.7 vs 37.5 %; *p* = 0.049). The similar observation was made for patients according to their mammographic breast density (Fig. 1c and d), as DFS was lower in patients with VLD breasts both in the intermediate and high risk NPI groups respectively (92.9 vs 76.7 %, 55.4 vs 17.2 %; *p* < 0.001).

Five known prognostic factors (ER status, PR status, HER2 status, breast density and the NPI) first underwent univariate analysis to assess their prognostic powers on our patient population. Only three HR values turned out to be statistically significant (HER2 status, NPI and VLD). Second, those three factors which retained the significance were put through Cox multivariate analysis. The values for both analyses are shown in (Table 3). Both HER2 and MBD proved to provide prognostic information independent of NPI.

**Table 3:** Hazard ratios of the prognostic factors in both the univariate and cox multivariate analysis

Prognostic factor	HR	*P*	95 % CI
Univariate analysis			
HER2 status	2.325	0.001	1.415–3.820
MBD (VLD)	1.986	0.004	1.238–3.187
NPI	2.295	<0.001	1.845–2.854
ER Status	0.995	0.986	0.596–1.662
PR Status	1.135	0.613	0.695–1.851
Multivariate analysis			
HER2 status	1.673	0.046	1.010–2.772
NPI	2.338	<0.001	1.872–2.920
MBD (VLD)	2.790	<0.001	1.724–4.516

### Incorporating HER2 and MBD into the NPI

NPI, MBD, and HER2 were selected in a final model to form the Kuopio-Nottingham Prognostic Index (K-NPI) with parameter estimates of 0.89 (SE, 0.113), 1.01 (SE, 0.246) and 0.51 (SE, 0.258), respectively. Since the parameter estimates of NPI and MBD were highly similar, the new model was calculated as the sum of those individual variables, in addition to + 0.5 for HER2 positivity. The optimal new cut-offs, obtained with the 0.632+ bootstrap method, were 5.1 and 5.9, the concordance index of the K-NPI was 0.872 as compared to 0.779 for the original NPI. As a result, patients in the K-NPI were now categorized into low-, intermediate-, and high-risk groups for values below 5.1, between 5.1 and 5.9, and higher than 5.9, respectively.

**Fig. 2:**
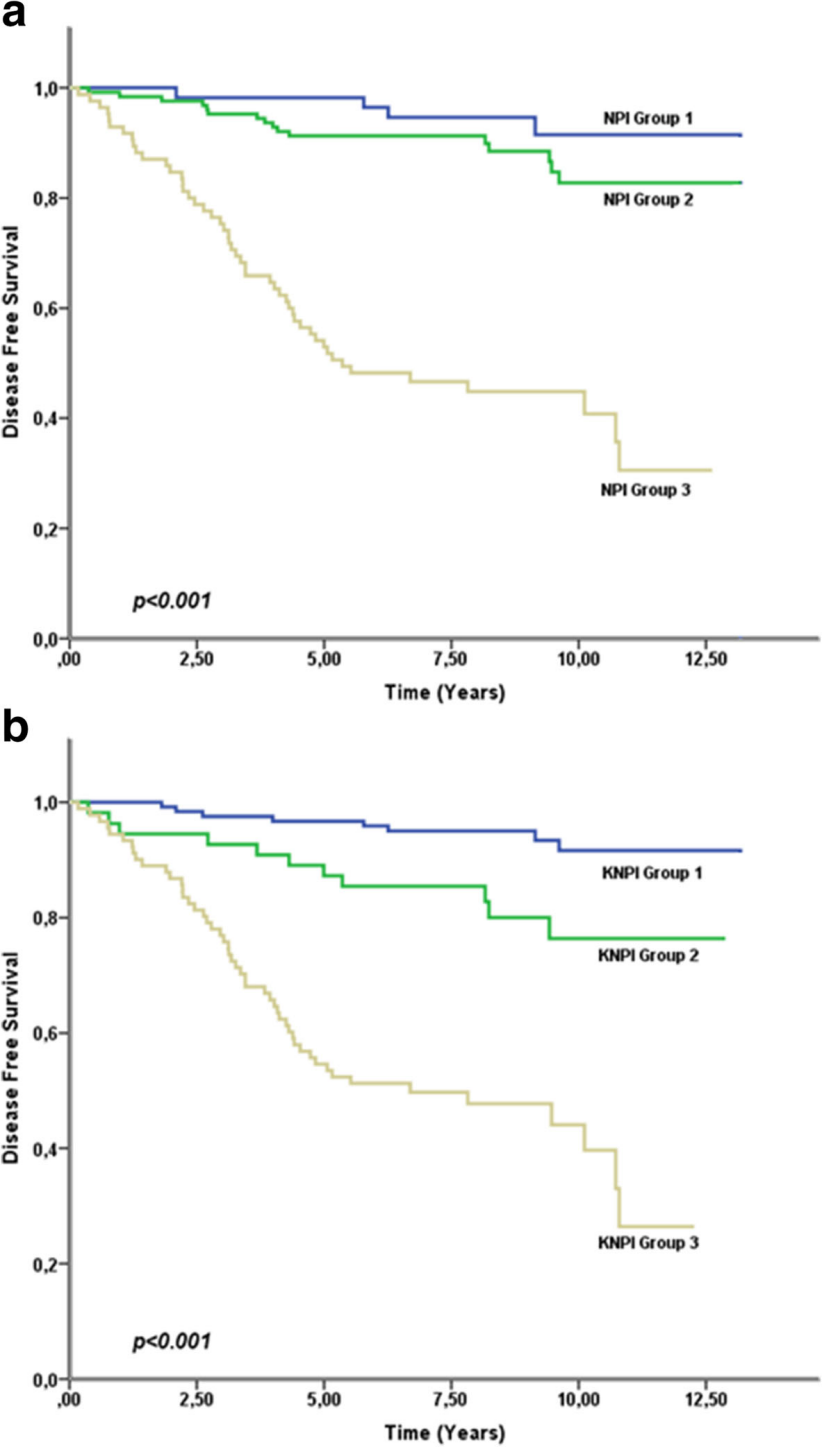
Graphs depicting DFS curves for risk groups of (**a**) the original NPI and (**b**) the newly coined KNPI

The classification of patients into the low, intermediate and high risk groups according to the K-NPI is compared to the original NPI in (Table 4) and the DFS of the new groups is illustrated in Fig. 2a and b. The new system managed to classify considerably less patients into the intermediate group (55 as compared to 127 in the original NPI model, *p* < 0.001) as demonstrated in (Table 5). Out of the 127 patients previously classified as intermediate risk, 66 were now classified as low risk, 16 as high risk and 45 remained as intermediate. With respect to DFS, 92.7 % (115/124), 80.0 % (44/55) and 45.1 % (41/91) were disease free in the low, intermediate and high risk groups according to the KNPI respectively at the end of the follow up period.

**Table 4:** Comparison between DFS in risk groups of the newly formed KNPI and the original NPI

	New Classification KNPI		Original NPI		*p*
Group	Patients (%)	DFS	Patients (%)	DFS	
Low risk	124 (45.9)	92.7 % (115/124)	58 (21.5)	91.4 % (53/58)	*<0.001*
Intermediate risk	55 (20.4)	80.0 % (44/55)	127 (47.0)	87.4 % (111/127)	
High risk	91 (33.7)	45.1 % (41/91)	85 (31.5)	42.4 % (36/85)	
C Index	*0.872*		*0.779*		

The distribution of patients into the newly formed low, intermediate and high risk groups of the Kuopio-Nottingham Prognostic Index with their respective Disease Free Survival, compared to the old categories of the original Nottingham Prognostic Index

**Table 5:** Distribution of the breast cancer patients (total *n* = 270) into different prognostic groups

	KNPI			
	Low risk	Intermediate risk	High risk	Total
NPI				
Low risk	58	0	0	*58*
Intermediate risk	66	45	16	*127*
High risk	0	10	75	*85*
*Total*	*124*	*55*	*91*	

Agreement: 0.513, *p* < 0.001

Comparison of the original Nottingham Prognostic Index with the Kuopio-Nottingham Prognostic Index

## Discussion

Breast cancer is a heterogeneous disease with varying phenotypes, genotypes, behaviours and responses to therapy. Adjuvant systemic treatments have helped to significantly decrease patient mortality. However, it is still difficult to evaluate which patients will benefit from adjuvant treatments and which patients will end up suffering from their toxicity [22, 23]. The principle finding of this study was that both HER2 status and very low mammographic breast density (VLD) proved to be independent of the classically used NPI and serve to improve its predictive ability. In our patient population, the original NPI classified a rather high proportion of patients into the intermediate risk group making it challenging to evaluate the need and benefit of adjuvant chemotherapy. With the new K-NPI, a considerable group of patients were moved from the intermediate to the low or high risk groups which might hold clinical significance in terms of adjuvant treatment decisions.

In line with our results, several studies have shown that HER2 status is a predictive factor independent of the NPI [24–26]. Although Van Belle et al. [26] managed to create a new prognostic classification system (dubbed the iNPI) by incorporating both HER2 and Progesterone status into the NPI, our results in contrast indicated that neither ER nor PR statuses were prognostically significant, which is in line with studies proposing that hormone receptors lose their prognostic power in the long term [27].

Previous studies have investigated the addition of several different factors to the NPI and whether those could serve to improve its predictive value in regards to patient prognosis [26, 28–31]. Mammographic breast density however has never been incorporated into a prognostic index before this trial, even though it is a routinely available, cost-free and easily interpreted parameter in patients with newly diagnosed breast cancer. Our results now show that MBD is a predictive factor independent of the NPI. Furthermore, it can be added to NPI simultaneously with HER2 status to give a synergistic advantage to its predictive ability, especially in the ubiquitous intermediate prognostic category of NPI. It can be clearly seen that MBD and HER2 status were major determinants in switching patients from the original NPI intermediate group to the new K-NPI low risk and high risk groups, density as shown in Table 6.

**Table 6:** The distribution of density categories and HER2 status in the patients who were in the original intermediate category of the NPI compared to their new distribution in the K-NPI

	Density	HER2
Low risk	VLD 3 (4.5 %)	HER2+ 25 (37.9 %)
	MID 63 (95.5 %)	HER2– 41 (62.1 %)
Intermediate risk	VLD 24 (53.3 %)	HER2+ 24 (53.3 %)
	MID 21 (46.7 %)	HER2– 21 (46.7 %)
High risk	VLD 16 (100 %)	HER2+ 11 (68.8 %)
	MID 0 (0 %)	HER2– 5 (31.3 %)

Our study is not without limitations. Our patient population is relatively small and we only had a limited number of triple negative cancers. And due to our patient selection criteria, our study had a higher percentage of HER2 positive patients than fully consecutive cohorts. Many of our patients have been treated with adjuvant therapies making it difficult to predict the exact role of the primary prognostic factors and how the treatments have affected the results. However, at the time of patient collection, the national guidelines in Finland were very similar to current guidelines. A notable exception was the addition of trastuzumab as a standard to HER2 positive patients in the year 2005, while before that trastuzumab was offered only for patients participating in the FinHer trial [21]. Furthermore, mammographic density was measured visually which may be considered less accurate by some, but we aimed to select a method that is easily reproducible in clinical practice and does not require the addition of expensive and sometimes complicated programs.

Another commonly used tool to evaluate patient outcome nowadays is the Adjuvant! Online prognostic index. It is an internet based computer programme providing 10-year prognosis predictions for early breast cancer patients. Its use has increased in recent years; however, its validation in different cohorts has not been as successful as its counterpart the NPI with many studies finding wide discrepancies between its reported predictions and actual survivals [32–34].

In the future, prognostic classification may benefit from newer methods such as microarray-based gene expression profiling [35]. Multigene signatures associated with prognosis have recently emerged and some are even commercially available [36]. Drukker et al. [37] showed a prognostic benefit by combining the 70-gene signature with the classical scoring systems. Nevertheless, these gene signatures carry many shortcomings, different multigene tests give different and variating results making their implementation into clinical practice difficult [38, 39]. This may be due to intratumoral genetic variation and heterogeneity in the microenvironment. Although these new markers may provide additional prognostic data, only a very limited number of patients could benefit from them due to the high costs of the tests. Thus, if we consider breast cancer as a global prolem, the classical clinical markers are still needed and new multigene tests should be considered complimentary and not a replacement for traditional parameters [40, 41]. Many breast cancer cases are diagnosed in the developing world where resources are scarce making those disadvantages particularly important, and that’s where the need stems for new, simple and easily available prognostic factors that are easy to interpret and can be easily combined with the classical clinicopathological scoring systems [23, 42]. HER2 status is nowadays measured routinely in most countries, and MBD can be easily acquired from the diagnostic mammograms, hence not requiring any extra time or money.

## Conclusions

In conclusion, our results show that for patients with early breast cancer MBD and HER2 status are indeed strong prognostic factors independent of the NPI. Furthermore, we were able to enhance the prognostic ability of NPI by the addition of HER2 status and breast density values into the newly coined K-NPI. This prognostic reclassification managed to significantly decrease the percentage of patients in the intermediate risk group, which serves to more reliably recognize those patients who are in the real higher risk group. Future work with larger patient populations, and with quantitative density measurement methods must be carried out to validate the clinical utility of our observations.

## Abbreviations

c-index: Concordance index
CISH: Chromogenic In situ hybridization test
DFS: Disease free survival
ER: Estrogen receptor
HER2: Human Epidermal Growth Factor Receptor 2
K-NPI: Kuopio-Nottingham Prognostic Index
MBD: Mammographic breast density
MID: Mixed density
NPI: Nottingham Prognostic Index
PR: Progesterone receptor
SE: Standard error
tdROC: Time-dependent receiver operating characteristic curve
VLD: Very low density
